# Risk Factors of Nephrolithiasis in a Tertiary Care Hospital in Rawalpindi: A Descriptive Cross-Sectional Study

**DOI:** 10.7759/cureus.26274

**Published:** 2022-06-24

**Authors:** Tayyab Mumtaz Khan, Muhammad Saad Anwar, Zubair Shafique, Fatima Kausar Nawaz, Muhammad Sikandar Karim, Danish Saifullah, Muhammad Zeshan Mehmood

**Affiliations:** 1 Surgery, Rawalpindi Medical University, Rawalpindi, PAK; 2 Neurology, AINeuroCare, Dallas, USA; 3 Internal Medicine, Sahiwal Medical College, Sahiwal, PAK; 4 Medicine, Shaikh Zayed Hospital, Lahore, PAK; 5 Research, California Institute of Neuroscience, Brooklyn, USA; 6 Medicine, Rawalpindi Medical University, Rawalpindi, PAK; 7 Surgery, Benazir Bhutto Hospital, Rawalpindi, PAK

**Keywords:** rawalpindi, cross-sectional, hospital, tertiary care, patients, admitted, risk factors, nephrolithiasis

## Abstract

Background

Nephrolithiasis (renal stones) is the most common urological disease. Its prevalence is high in every part of the world. Several factors lead to renal stone formation. In Pakistan, nephrolithiasis prevalence is also high as Pakistan is located in a region which is known as the salt belt. However, nephrolithiasis and its possible risk factors are under-researched in Pakistan.

Objective

This study aims to identify the risk factors for nephrolithiasis among admitted patients with renal stones. This may lead to a reduction in renal stone incidence and its allied complications by the prevention of risk factors that would have a major role in renal stone formation.

Material and methods

This descriptive cross-sectional study was performed among the 143 admitted patients with renal stones in the urology ward of Benazir Bhutto Hospital, Rawalpindi, for approximately six months from November 2021 to April 2022. Non-probability convenient sampling and developed inclusion and exclusion criteria were used for the recruitment of patients. After elaborating on the objectives, the study data were collected by interviewers through a self-structured questionnaire. Descriptive analysis was carried out using SPSS version 25.0 (IBM Corp., Armonk, NY).

Results

Nephrolithiasis was more prevalent among patients who had an age group range of 15-30 years (47.55%), male gender (56.65%), illiterate educational status (53.14%), lower socioeconomic status (66.43%), inadequate intake of water (61.53%), used tap water (56.64%), a habit of daily vegetable intake (65.04%), sedentary lifestyle (51.74%), family history of renal stones (57.34%), no diabetes mellitus (62.94%), no hypertension (52.45%), and overweight (48.23%).

Conclusion

In brief, the age group of 15-30 years, male gender, illiteracy, lower socioeconomic status, insufficient water intake, tap water, high vegetables, inactive lifestyle, family history of nephrolithiasis, and a high BMI all increase the risk of nephrolithiasis.

## Introduction

Nephrolithiasis (renal stones) is a urological condition in which stones are formed inside the kidneys when the precipitation of crystals from urine takes place. Precipitation of crystal inside the urinary tract could take place either in excess presence of the stone forming material or deficiency of the substances that prevent stone formation [1,2]. Patients with nephrolithiasis could be asymptomatic. However, they could present with frequent symptoms such as flank pain, nausea, vomiting, urinary tract infection, or urinary tract obstruction [3].

Nephrolithiasis is the third most prevalent condition globally; about 15% of its patients belong to western countries. It affects nearly one in every 13 women and one in every seven men worldwide [3,4]. In Pakistan, the risk of renal stones is also high because Pakistan is present in the region which is known as the stone belt region, and other countries in this region include India, Burma, Egypt, Thailand, Sudan, Indonesia, and the Philippines. These countries report a high frequency of urinary tract stones [5].

Renal stones are associated with a significant expense to society because of their great prevalence and high frequency of repeated occurrence. Furthermore, renal stones could lead to several complications, such as decreased renal performance, hydronephrosis, urinary tract infection, and eventually kidney failure [6]. So, it is agreeable that renal stones have an adverse influence on the health and financial lives of their patients.

Socio-demographic elements such as age, gender, educational status, and socioeconomic class play a role in the formation of kidney stones. Furthermore, other risk factors that contribute to the development of renal stones include inadequate water intake, water intake without filtration, a high intake of raw vegetables, daily tea intake, inactive lifestyle, history of renal stone in the family, smoking, high serum level of lipids, diabetes mellitus, and hypertension [6-9].

In Pakistan, nephrolithiasis is under-studied and people are not truly familiar with its risk factors, which is why its incidence is high among the natives of Pakistan. Likewise, the underdeveloped health care system and restricted sources of education about diseases further aggravate the health-related issues. In the presence of little information about the risk factors in Pakistan, this study aims to identify the risk factors for nephrolithiasis among admitted patients with nephrolithiasis at the urology department of Benazir Bhutto Hospital, Rawalpindi. By recognizing the risk factor of renal stones, we would be able to educate the community that they could avoid renal stones, and this could lead to support for the health care system as well, by decreasing the patients’ overload on hospitals.

## Materials and methods

We conducted this descriptive cross-sectional study on diagnosed patients with nephrolithiasis who were admitted to the urology department of the Benazir Bhutto Hospital, Rawalpindi, Pakistan, for approximately six months from November 2021 to April 2022. One hundred and forty-three patients were recruited through a non-probability convenient sampling technique and established inclusion and exclusion criteria. Only admitted patients aged 15 years or older, diagnosed with nephrolithiasis, and willing to participate in the study were included, while admitted patients aged less than 15 years, suspected of having nephrolithiasis, and unwilling to participate were excluded. Informed consent was acquired from all patients, and the aims of the study were explained to them before the interview. Interviewers conducted the interviews of patients and collected information by filling in the self-designed questionnaire about the socio-demographic profile of participants, which included age group (15 to 30 years, 31 to 45 years, or above 45 years), gender (male or female), educational status (illiterate, middle, matric or above), socioeconomic class based on monthly income (lower=up to 25,000 Pkr per month, middle=26,000 to 55,000 Pkr, and upper=above 55,000 Pkr), and possible risk factors of nephrolithiasis such as quantity of water intake based on daily water intake (adequate = 8 or more than 8 glasses or inadequate = less than 8 glasses), type of water intake (filtered or tap water), daily vegetable intake (yes or no), lifestyle based on 20 minute daily walk (yes=active or no=sedentary), family history of renal stone (yes or no), diabetes mellitus presence (yes or no), hypertension (yes or no), and nutritional status based on BMI (normal if BMI=18.50-24.90, underweight if BMI=less than 18.50, overweight if BMI=25.00-29.90, and obese if BMI=30.00 or above). We applied the WHO classification, which is based on BMI, for the evaluation of the nutritional status. Tools that were used for BMI calculation included a weight machine to measure weight in kilograms and a measuring tape for measuring height in meters. Calculation of the BMI was done through measurement of height in meters and weight in kilograms of patients via the following formula (BMI=weight/height^2^).

After the completion of the data collection, data analysis was performed by using SPSS version 25.0. For the calculation of the frequencies and percentages of the qualitative variables and the means of quantitative variables, descriptive analysis was carried out.

## Results

Out of 143 participants, 81 (56.65%) were males and 62 (43.35%) were females. The means of age and BMI for the study population were 38.76 with a standard deviation (SD) of ±16.34 and 24.7 with an SD of ±7.81, respectively.

Table 1 indicates that nephrolithiasis incidence was more common among patients who had an age group of 15-30 years, male gender, lower educational status, and low socioeconomic status as well in comparison to patients who had other older age groups like 31-45 years, above 45 years, female gender, higher educational status, and middle or upper socioeconomic classes, respectively.

**Table 1:** Socio-demographic characteristics of the study population.

Variable	Frequency	Percentage
Age group in years		
15-30	68	47.55%
31-45	41	28.68%
Above 45	34	23.77%
Gender		
Male	81	56.65%
Female	62	43.35%
Educational status		
Illiterate	76	53.14%
Middle	42	29.37%
Matric or above	25	17.49%
Socioeconomic class		
Lower	95	66.43%
Middle	40	27.98%
Upper	08	5.59%

Table 2 shows that nephrolithiasis incidence was more prevalent among the patients who had inadequate intake of water, used tap water, took daily vegetables in the diet, sedentary lifestyle, family history of renal stones, had no diabetes mellitus, no hypertension, and had high BMI as compared to patients who had adequate intake of water, used filtered water, did not take daily vegetables in the diet, active lifestyle, no family history of renal stones, diabetes mellitus, hypertension, and low BMI, respectively. However, a significant number of patients with nephrolithiasis had diabetes mellitus and hypertension.

**Table 2:** Frequency and percentages of the risk factors in the study population.

Variables	Frequency	Percentage
Daily water intake		
Adequate	55	38.46%
Inadequate	88	61.53%
Type of water intake		
Filtered	62	43.36%
Tap	81	56.64%
Daily vegetable intake		
Yes	93	65.04%
No	50	34.96%
Life style		
Active	69	48.26%
Sedentary	74	51.74%
Family history of renal stones		
Yes	82	57.34%
No	61	42.66%
Diabetes mellitus		
Yes	53	37.06%
No	90	62.94%
Hypertension		
Yes	68	47.55%
No	75	52.45%
Nutritional status		
Underweight	14	9.80%
Normal weight	50	34.97%
Overweight	69	48.23%
Obese	10	7.00%

## Discussion

The findings of this research study have presented invaluable information about the risk factors of a common urological problem, which is nephrolithiasis.

In the first step of data analysis, we assessed the role of socio-demographic characteristics of the study population in the formation of renal stones. Nephrolithiasis was more prevalent (47.55%) among the age group with a range of 15-30 years. A study that was also conducted in Pakistan showed similar findings with the highest prevalence of renal stones in an almost similar age group of 18-30 years [2]. An Indian study reported conflicting findings with the maximum renal stone incidence in the age group of 45-50 years [6]. The higher frequency of nephrolithiasis in the male gender (56.65%) in these study results was also supported by a study that was carried out in Iran. However, an Indian study noted the high prevalence of renal stones in the female gender [6,10]. The formation of renal stones due to low educational status was recorded in another study in China as well as this study [7]. This study also suggested that patients with a lower socioeconomic class had a higher incidence of nephrolithiasis, and this statement was also endorsed by another study [3]. Here, we could say that it is acceptable that socio-demographic factors have a role in the development of renal stones.

In the next and final step of the analysis of the data, frequencies and percentages of other potential risk factors for nephrolithiasis among the patients with nephrolithiasis were noted. Renal stones were more prevalent in patients who had an inadequate intake of water and had used tap water. Similar results regarding the quantity of water intake and quality of water were described in several studies that were executed in different parts of the world [3,11,12]. High consumption of vegetables could also lead to renal stone production, and it was also described in other studies [8]. A sedentary lifestyle predisposes people to nephrolithiasis and this statement of the current research was backed by another study [13]. Family history of nephrolithiasis could cause a high incidence of it and it was also documented in other research projects identical to this study [6,8]. Renal stones were more prevalent in the non-diabetic group in our study population. However, another study proposed that diabetes mellitus puts its patients at the risk of renal stone formation [14]. This study presented a high frequency of renal stones among patients with normal blood pressure, in contrast to a study that reported hypertension as a risk factor for renal stone generation [7]. The high prevalence of renal stones in non-diabetic and normotensive people could be due to the relatively young age of the study population as in other studies; diabetes mellitus and hypertension have been recorded as significant risk factors for renal stones as mentioned above. High BMI, which means overweight or obesity, was noted to increase nephrolithiasis incidence in study participants, and a similar observation was also made by some other studies [2,14].

Besides socio-demographic elements, there were also other factors that increased renal stone formation, as discussed above. Although this study has provided evidence-based data about the risk factors of renal stones, it has some limitations as well, such as its cross-sectional design could not explain how these risk factors cause the renal stone formation and it was conducted among patients who belonged to a particular region of Pakistan, so, in other regions, nephrolithiasis risk factors could be different. Therefore, this research recommends further research with other study designs and in different areas is needed to find out the way renal stones are caused by these factors and what the risk factors are in other areas, whether those are the same or different. Health authorities should make people educated about the risk factors of renal stones and how they can avoid them to bring down the high incidence of nephrolithiasis. 

This research recommends that people should drink enough water after its proper filtration. People should consume green, leafy vegetables in the required quantity. People should avoid a sedentary lifestyle and should be engaged in some physical activity to keep body fat in the normal range. All these strategies would help them to avoid nephrolithiasis.

## Conclusions

Our study results have suggested that nephrolithiasis occurs in the age group of 15-30 years, in the male gender, with lower educational status and lower socioeconomic class. The major risk factors associated with the development of nephrolithiasis are inadequate fluid intake, drinking tap water, high vegetable consumption, sedentary lifestyle, family history of renal stones, and high BMI. A relatively greater number of patients with renal stones did not have diabetes mellitus and hypertension. Healthcare systems should educate people about the risk factors that predispose people to nephrolithiasis. Health authorities could increase people's education level about risk factors for nephrolithiasis and its associated complications through public service announcements, regular health fairs, newsletters, and mass media campaigns. By increasing the education level of the public about the risk factors of renal stones and their preventive measures, the nephrolithiasis incidence could be brought down, which would surely improve the quality of life of the general public that could suffer from renal stones. Furthermore, it would also prevent renal stone-associated complications and would support the underdeveloped and resource-deficient healthcare system.
